# Optimization of Therapeutic modRNA Delivery to the Lung for Prevention of Pulmonary Fibrosis

**DOI:** 10.3390/pharmaceutics18070868

**Published:** 2026-07-16

**Authors:** Gayatri Mainkar, Magdalena M. Zak, Matteo Ghiringhelli, Jimeen Yoo, Matthew Adjmi, Keerat Kaur, Lior Zangi

**Affiliations:** 1 Cardiovascular Research Institute, Icahn School of Medicine at Mount Sinai, New York, NY 10029, USA; gaya.mainkar@icahn.mssm.edu (G.M.); magdalena.zak@mssm.edu (M.M.Z.); ghiringhelli.matteo@mssm.edu (M.G.); jimeenyoo@gmail.com (J.Y.); matthew@adjmi.com (M.A.); keerat08@gmail.com (K.K.); 2 Department of Genetics and Genomic Sciences, Icahn School of Medicine at Mount Sinai, New York, NY 10029, USA; 3 Department of Stem Cell Biology and Regenerative Medicine, Icahn School of Medicine at Mount Sinai, New York, NY 10029, USA

**Keywords:** mRNA therapeutic, mRNA delivery, lipid nanoparticle-encapsulated mRNA, lung fibrosis

## Abstract

**Background/Objectives:** Pulmonary fibrosis is a progressive and fatal disease characterized by excessive extracellular matrix deposition and irreversible lung remodeling. Although modified mRNA (modRNA) therapeutics offer a promising strategy for regulating disease-driving pathways, effective pulmonary delivery remains challenging due to the inherent liver tropism of conventional lipid nanoparticles (LNPs). This study aimed to establish an optimized platform for lung-selective modRNA delivery and therapeutic screening for pulmonary fibrosis. **Methods:** A panel of charge-modified LNP formulations was evaluated in vivo for pulmonary tropism following systemic administration of luciferase (Luc) modRNA. Administration routes, biodistribution in healthy and bleomycin (BLM)-induced fibrotic lungs, and endogenous microRNA (miRNA)-mediated de-targeting strategies were assessed. Candidate antifibrotic modRNAs targeting the transforming growth factor-beta (TGF-β) signaling pathway were subsequently evaluated in normal human lung fibroblasts (NHLFs). **Results:** Among the formulations tested, 50% DOTAP MC3 LNPs demonstrated the most favorable balance of pulmonary transfection, physicochemical properties, and limited off-target expression. Intravenous (IV) administration achieved robust lung expression with a superior safety profile compared with intratracheal (IT) delivery. Importantly, pulmonary biodistribution was preserved in BLM-induced fibrotic lungs despite extensive tissue remodeling. Incorporation of miR-122 recognition sites further enhanced selectivity, resulting in 94.5% of total transgene expression being localized to the lungs while substantially reducing residual hepatic expression. In vitro screening identified dominant-negative TGF-β receptor II (DNTGFBR2) modRNA as a potent inhibitor of TGF-β-induced fibrotic activation, significantly suppressing α-SMA and CTGF expression. **Conclusions:** These findings establish a comprehensive platform for pulmonary modRNA therapeutic development by integrating lung-selective LNP engineering, optimal systemic delivery, miRNA-mediated de-targeting, and therapeutic payload screening. This strategy provides a foundation for the development of targeted RNA therapies for pulmonary fibrosis and other organ-specific diseases.

## 1. Introduction

Pulmonary fibrosis is a debilitating, chronic, progressive disease widely thought to be driven by recurring injuries to the alveolar epithelium that trigger a maladaptive wound-healing cascade [1,2]. This aberrant response causes the hyperactivation and differentiation of resident fibroblasts into matrix-secreting myofibroblasts, leading to excessive extracellular matrix (ECM) deposition [3]. The resulting remodeling of the lung parenchyma impairs gas exchange and can ultimately lead to respiratory failure. Current FDA-approved small-molecule therapies, such as pirfenidone and nintedanib, merely slow disease progression without halting it, and their clinical utility is further limited by systemic adverse effects including liver toxicity and cardiac distress [4,5,6]. Consequently, there remains a need for therapeutic strategies capable of selectively disrupting profibrotic pathways within lung tissue while minimizing systemic toxicity.

Protein replacement therapies have emerged as promising approaches for restoring protective cellular functions disrupted during disease progression. Nucleoside-modified mRNA (modRNA) offers several advantages over other protein replacement approaches, such as DNA-loaded viral vectors and recombinant proteins, by avoiding pre-existing viral immunity, packaging size limitations, and genomic integration risks while enabling rapid, dose-controlled protein expression with a therapeutically relevant biological half-life [7,8,9]. In contrast to RNA modalities such as small interfering RNAs (siRNAs) and antisense oligonucleotides (ASOs), which primarily suppress endogenous gene expression, modRNA is particularly well suited for gain-of-function applications requiring transient therapeutic protein expression. Although circular RNAs and self-amplifying RNAs can achieve prolonged protein expression, their extended persistence may not be desirable for applications requiring precise temporal control of signaling pathways [10]. The transient nature of modRNA, combined with its favorable safety profile and high translational efficiency, therefore makes it an attractive platform for delivering therapeutic proteins involved in tissue repair and regeneration.

To achieve intracellular translation in vivo, lipid nanoparticles (LNPs) are considered the clinical gold standard for the delivery of modRNA therapeutics [11,12,13]. However, conventional LNP formulations exhibit a strong intrinsic tendency to accumulate in the liver, largely through interactions with apolipoprotein E (ApoE), which remains a major barrier to effective pulmonary delivery [14]. Recent studies have demonstrated that modifying LNP composition can partially redirect biodistribution toward the lungs, highlighting the promise of rational nanoparticle engineering for pulmonary RNA therapeutics [15,16]. Nevertheless, therapeutic RNA delivery to the lungs faces several additional biological barriers. Following either systemic or local administration, nanoparticles must avoid extracellular RNase-mediated degradation, penetrate the mucus layer lining the respiratory epithelium, evade rapid uptake by alveolar macrophages, undergo efficient cellular internalization, and successfully escape the endosomal compartment before lysosomal degradation occurs [17]. Collectively, these barriers substantially reduce the fraction of delivered RNA that ultimately reaches the cytoplasm and is available for translation, making efficient pulmonary delivery considerably more challenging than delivery to highly perfused organs such as the liver.

This study aims to establish a platform for screening candidate therapies for pulmonary fibrosis by optimizing lung-selective modRNA delivery and expression. Previous studies have demonstrated that introducing permanently positively charged lipids into LNPs alters plasma protein corona formation, imparting intrinsic lung tropism [18,19]. We therefore compared multiple charge-modified LNP formulations to identify one that achieved robust and selective pulmonary transfection. Selecting the optimal administration route is equally critical, as it dictates systemic circulation kinetics versus localized organ exposure [20]. While direct airway delivery concentrates vehicle exposure within respiratory tissues, it risks triggering localized epithelial damage; conversely, systemic intravenous (IV) circulation may provide a safer exposure profile but requires highly selective nanoparticles capable of overcoming hepatic clearance.

The accumulation of collagen bundles, myofibroblast proliferation, and restricted perfusion in the fibrotic tissue characteristic of pulmonary fibrosis may create physical barriers that impede nanoparticle diffusion through the interstitium and limit cellular uptake relative to healthy lung tissue [21]. Therefore, evaluating whether a fibrotic microenvironment alters LNP delivery in vivo is a prerequisite for validating candidate LNP formulations.

While charge-engineered LNPs enhance lung-tropic delivery, residual off-target translation in vascularized peripheral organs can be further eliminated by leveraging endogenous microRNA (miRNA)-mediated translational repression to maximize selectivity. MiRNAs exhibit diverse expression profiles across tissues and organs, and previous studies have demonstrated that these intrinsic differences can be leveraged to restrict transgene expression to selected tissues [22]. Incorporating tissue-specific miRNA recognition elements into therapeutic modRNAs therefore provides an additional layer of control that complements organ-selective nanoparticle delivery.

Finally, once an optimized delivery platform has been established, in vitro screening strategies are required to prioritize candidate therapeutic payloads before progressing to in vivo evaluation. Because the canonical transforming growth factor-beta (TGF-β) signaling axis is a principal driver of pulmonary fibrosis, an in vitro platform designed to measure the suppression of downstream targets within this cascade was utilized to evaluate candidate payloads [23]. Collectively, these complementary approaches establish a lung-selective modRNA delivery platform that can accelerate the development of targeted RNA therapeutics for pulmonary fibrosis.

## 2. Materials and Methods

### 2.1. Synthesis and Characterization of modRNA-LNP Complexes

ModRNAs were generated by in vitro transcription from linearized DNA templates using T7 RNA polymerase, as described before [24]. Transcripts were co-transcriptionally capped using CleanCap AG, and uridine was replaced with N1-methylpseudouridine-5′-triphosphate. Lipid 5 LNPs were formulated using Lipid 5, DSPC, cholesterol, and DMG-PEG2000 at a molar ratio of 50:10:38.5:1.5; iPLNPs using 9A1P9, DDAB, cholesterol, and DMG-PEG2000 at 60:30:40:0.4; 50% DOTAP 5A2SC8 LNPs using 5A2SC8, DOPE, cholesterol, and DMG-PEG2000 at 5:15:30:3; and 50% DOTAP MC3 LNPs using DLin-MC3-DMA, DSPC, cholesterol, and DMG-PEG2000 at 50:10:38.5:1.5. All lipids were purchased from Avanti Polar Lipids or Echelon Biosciences. LNPs were formulated by microfluidic mixing (Cytiva, Hauppauge, NY, USA) of the lipid and RNA phases and characterized by dynamic light scattering (Anton Paar, Graz, Austria) to determine hydrodynamic diameter and polydispersity index (PDI). Encapsulation efficiency (EE) was quantified using the Quant-iT RiboGreen RNA Assay (Thermo Fisher Scientific, Waltham, MA, USA) according to the manufacturer’s instructions.

### 2.2. In Vivo modRNA Delivery and Biodistribution Analysis

Male C57BL/6 mice were administered modRNA-loaded LNPs (25 µg modRNA/mouse) via IV (50 µL/mouse), IP (100 µL/mouse), or IT (50 µL/mouse) delivery as indicated for each experiment. At designated time points, animals were euthanized, and organs were harvested for ex vivo biodistribution analysis. Luciferase expression was quantified using an IVIS Spectrum imaging system (PerkinElmer, Shelton, CT, USA), and bioluminescence signals were measured and normalized as described in the corresponding figure legends. Organ distribution profiles were used to compare LNP formulations, administration routes, and the effects of pulmonary fibrosis and miRNA-mediated de-targeting on modRNA expression.

### 2.3. Flow Cytometric Analysis

To assess pulmonary cell transfection, Cre modRNA-loaded LNPs were administered to Ai14 reporter mice. At the indicated time points, lungs were perfused, harvested, mechanically dissociated, and enzymatically digested in a collagenase-, DNase I-, and hyaluronidase-containing digestion buffer for 1 h at 37 °C with agitation. The resulting cell suspension was filtered through a 100 μm cell strainer, washed with PBS, and centrifuged to obtain a single-cell suspension. Red blood cells were lysed using red blood cell lysis buffer, and cells were subsequently resuspended in FACS buffer. Cells were stained with fluorophore-conjugated antibodies against CD31, CD45 and EpCAM (BioLegend, San Diego, CA, USA) for 20 min at 4 °C in the dark. Antibodies were used at a 1:100 dilution. Following staining, cells were washed and resuspended in FACS buffer containing a viability dye to exclude dead cells prior to analysis. Samples were acquired on a flow cytometer (Cytek Aurora, Fremont, CA, USA) and analyzed using FlowJo v11 software (BD Biosciences, Milpitas, CA, USA).

### 2.4. Bleomycin-Induced Pulmonary Fibrosis Model and Histological Assessment

Pulmonary fibrosis was induced by an IT instillation of bleomycin (BLM; 2.5 mg/kg) on day 0. Disease progression was monitored longitudinally by body weight measurements, and lungs were collected at the indicated experimental endpoints for downstream analyses. For histological assessment, mice were perfused with PBS prior to tissue collection. Lungs were harvested, fixed, and cryosectioned for staining using the Trichrome Stain Kit (Sigma-Aldrich, Saint Louis, MO, USA) according to the manufacturer’s instructions. Total lung collagen content was quantified using a hydroxyproline assay (Sigma-Aldrich). For safety profiling, whole blood was collected via cardiac puncture into anticoagulant-coated and serum collection tubes. Samples were centrifuged to separate plasma and serum, which were subsequently submitted to the Mount Sinai Histology and Pathology Core for hematological and serum chemistry analyses.

### 2.5. In Vitro Fibrosis Model and Gene Expression Analysis

Primary normal human lung fibroblasts (NHLFs; Lonza, Portsmouth, NH, USA) were cultured according to the manufacturer’s instructions. Cells were seeded in 6-well plates and transfected with modRNAs using JetMessenger (Polyplus, Illkirch, France) according to the manufacturer’s protocol, with 2 μg of modRNA administered per well. Twenty-four hours after transfection, cells were stimulated with transforming growth factor-beta 1 (TGF-β1; PeproTech, Cranbury, NJ, USA) at a final concentration of 5 ng/mL to induce a profibrotic phenotype. Forty-eight hours following TGF-β1 stimulation, cells were collected for qPCR analysis or fixed for IF staining. qPCR was performed using primers for α-SMA and CTGF (IDT, Coralville, IA, USA). Relative gene expression was calculated using the ΔΔCt method and normalized to GAPDH expression. For IF analysis, cells were stained for α-SMA (Abcam, Waltham, MA, USA) and counterstained with DAPI prior to imaging on a Zeiss fluorescence microscope. Quantification of the α-SMA-positive area was performed using Fiji (ImageJ, v1.54t) software.

### 2.6. Use of Artificial Intelligence During Manuscript Preparation

Generative artificial intelligence (ChatGPT, OpenAI GPT-5.5) was used during manuscript preparation to assist with language refinement, restructuring of the text, and improving the clarity and organization of the manuscript. All scientific content, data interpretation, conclusions, and final editorial decisions were developed and verified by the authors. The authors critically reviewed and edited all AI-generated text and take full responsibility for the content of the manuscript.

## 3. Results

### 3.1. Comparative In Vivo Screening of LNP Formulations for Pulmonary Tropism

**Figure 1:**
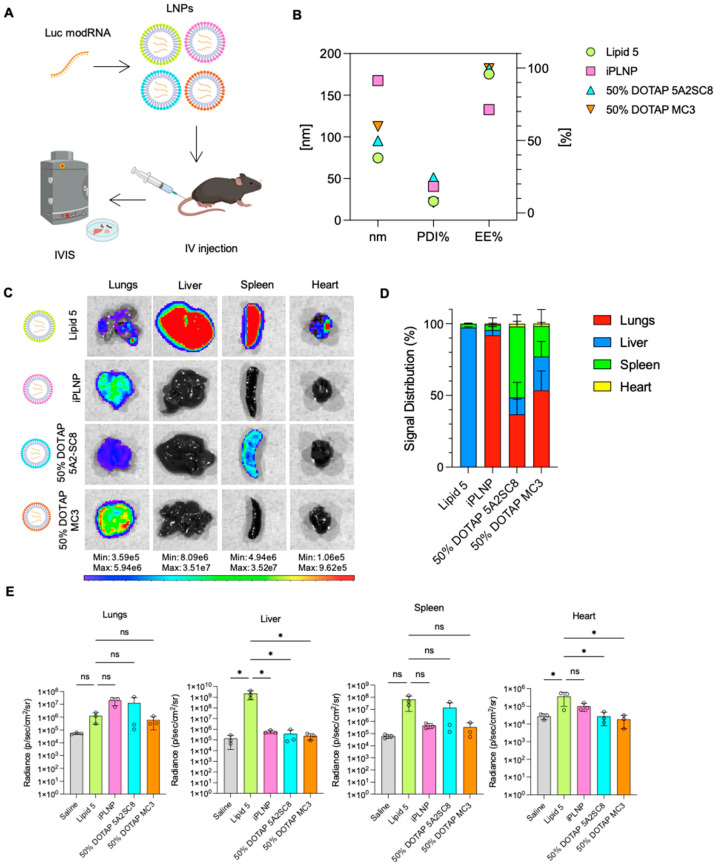
Characterization and evaluation of systemic organ tropism of various modRNA-loaded LNP formulations. (**A**). Schematic workflow of Luc modRNA encapsulated in different LNP formulations administered via IV injection, followed by ex vivo imaging using an IVIS. (**B**). Physicochemical features of candidate LNP formulations including size (nm), PDI (%) and EE (%). (**C**). Representative ex vivo IVIS images of lungs, liver, spleen, and heart from mice injected with each type of LNP. Images are shown using fixed, organ-specific intensity scales. (**D**). Relative Luc expression across organs per treatment cohort, expressed as the percentage of the total normalized bioluminescence signal measured across all collected organs. Data were normalized to the corresponding saline control. (**E**). Absolute quantification of ex vivo tissue bioluminescence (p/s/cm^2^/sr) across organ groups. Statistical significance was determined using one-way ANOVA followed by Tukey’s multiple comparisons test. Data are presented as mean ± SD (*N* = 3). ns, not significant; * *p* < 0.05. Created in BioRender. Mainkar, G. (2026) https://BioRender.com/3fm145i (**A**).

To evaluate candidate LNPs capable of bypassing hepatic sequestration for lung-targeted applications, a diverse panel of established positively charged formulations was selected for comparative in vivo screening (Figure 1A). The panel included a formulation utilizing Lipid 5, chosen as a control due to its known high multi-tissue transfection efficiency and neutral charge [25]. This control was evaluated against three previously reported positively charged LNP formulations composed of distinct ionizable and helper lipid combinations (Figure S1). These included a formulation featuring the permanently cationic dimethyldioctadecylammonium bromide (DDAB) paired with an ionizable phospholipid (iPLNP) shown to enhance endosomal escape, as well as two formulations supplemented with 50% permanently cationic 1,2-dioleoyl-3-trimethylammonium-propane (DOTAP) using either 5A2-SC8, a highly degradable dendrimer, or the more clinically validated lipid DLin-MC3-DMA (MC3) (Figure S1) [15,16]. Upon synthesis, each LNP demonstrated an acceptable polydispersity index (PDI); however, iPLNP had a relatively larger hydrodynamic size and lower encapsulation efficiency (EE) than the others (Figure 1B). Following IV delivery of the luciferase (Luc) modRNA encapsulated in each LNP to mice, ex vivo bioluminescence imaging revealed distinct biodistribution profiles; the Lipid 5 LNP remained restricted to the liver as expected, whereas iPLNP and both 50% DOTAP candidates had higher tropism to the lungs (Figure 1C,D). While iPLNP mediated the highest absolute lung expression, its lower EE may have limited translational scalability (Figure 1B,E). Conversely, 50% DOTAP 5A2-SC8 LNP generated excessive off-target accumulation within the spleen (Figure 1C,D). Ultimately, 50% DOTAP MC3 LNP emerged as the optimal vehicle, combining favorable physicochemical properties with robust pulmonary transfection and minimal off-target signals in the liver, spleen and heart tissue (Figure 1B–D).

To confirm the broad transfection capacity of the lead candidate within the lungs, Cre recombinase (Cre) modRNA encapsulated in 50% DOTAP MC3 LNPs was delivered to Cre-dependent fluorescent reporter mice (Figure S2A). One week after injection, lungs were harvested for fluorescence-activated cell sorting (FACS) analysis, which showed extensive transfection across endothelial, epithelial, and immune cells, with the percentage of transfected cells quantified for each of these pulmonary cell populations (Figure S2B,C). Collectively, comparative in vivo screening identified 50% DOTAP MC3 LNPs as an effective, broad-spectrum carrier for pulmonary gene delivery.

### 3.2. Evaluation of Administration Routes for Pulmonary Delivery

**Figure 2:**
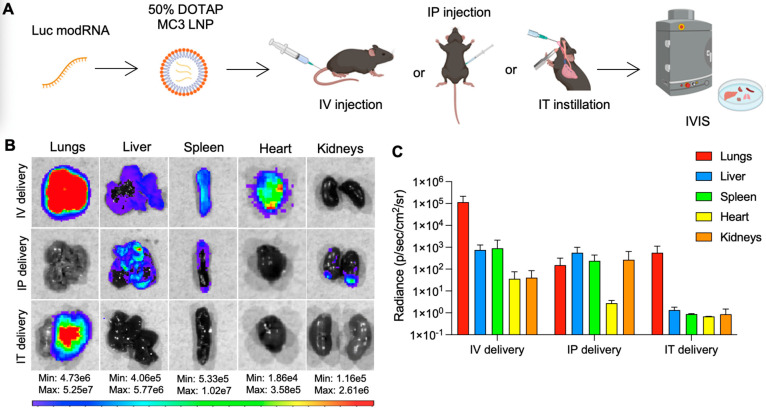
Evaluation of different administration routes for lung-targeted 50% DOTAP MC3 LNP delivery. (**A**). Schematic workflow of Luc modRNA encapsulated in 50% DOTAP MC3 LNPs administered via three distinct routes, IV injection, IP injection, or IT instillation, followed by ex vivo bioluminescence imaging using an IVIS. (**B**). Representative ex vivo IVIS images of harvested organs showing Luc expression patterns across the three delivery routes. Images are shown using fixed, organ-specific intensity scales. (**C**). Quantitative comparative analysis of ex vivo tissue bioluminescence (p/s/cm^2^/sr) within each organ group normalized to saline as a function of the administration route (*N* = 5, data presented as mean ± SD). Created in BioRender. Mainkar, G. (2026) https://BioRender.com/3fm145i (**A**).

Next, different delivery routes were compared to determine the ideal pathway for lung-targeted gene expression using the 50% DOTAP MC3 LNPs. Luc modRNA was administered to mice via IV injection, IP injection, or IT instillation, followed by ex vivo bioluminescence imaging analysis 24 h post-administration (Figure 2A). IV delivery resulted in the highest absolute lung expression as well as a shift in biodistribution from the liver to the lungs, although a baseline level of hepatic accumulation was still retained (Figure 2B,C). IP delivery resulted in a diffuse signal primarily localized to abdominal organs, such as the liver, spleen, and kidneys (Figure 2B,C). In contrast, IT delivery concentrated the bioluminescence signal almost exclusively within the lungs, yielding negligible off-target leakage (Figure 2B,C).

The rate of mortality in the IT cohort, however, was very high, and lungs harvested from these mice exhibited signs of severe tissue damage, likely triggered by an acute inflammatory response from the alveolar tissue being directly exposed to the charged LNPs (Figure S3D). In contrast, mice receiving IV or IP administration exhibited no overt signs of treatment-related toxicity. Comprehensive safety profiling demonstrated that IV administration maintained serum liver enzyme levels and circulating immune cell populations within physiological ranges (Figure S3A–C), supporting the favorable tolerability of this route. Collectively, these findings indicate that although IT administration achieves highly localized pulmonary delivery, it is associated with substantial local toxicity, whereas IV administration provides an improved balance between pulmonary transfection efficiency and systemic tolerability.

### 3.3. Evaluation of LNP Delivery Efficacy in a BLM-Induced Pulmonary Fibrosis Model

**Figure 3:**
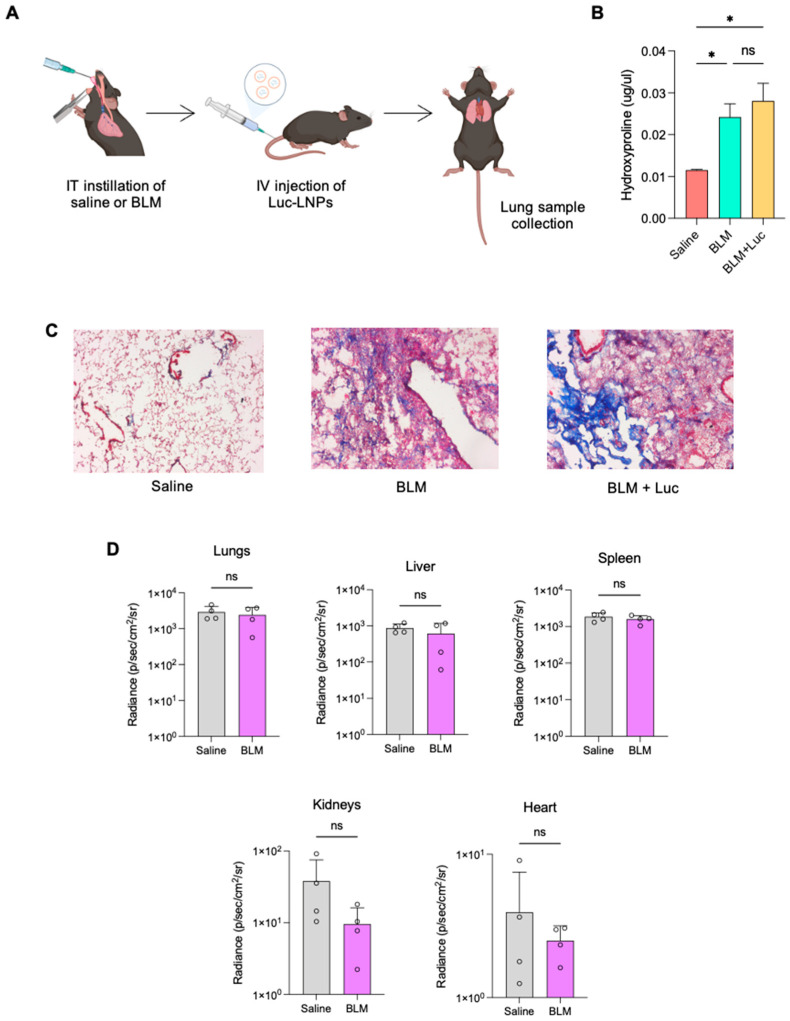
Evaluation of biodistribution and impact on fibrotic phenotype of 50% DOTAP MC3 LNPs. (**A**). Schematic workflow outlining IT administration of BLM at day 0 to establish lung injury, followed by IV delivery of 50% DOTAP MC3 LNPs carrying Luc at day 10. (**B**). Hydroxyproline assay quantifying total collagen content within lung samples collected on day 21. (**C**). Representative high-magnification images of histological sections of lung parenchyma stained with Masson’s trichrome. (**D**). Quantification of ex vivo tissue bioluminescence (p/s/cm^2^/sr) across organs harvested at day 11 from control and injured cohorts normalized to saline. No significant differences in radiance were observed between saline-treated and BLM-treated mice, demonstrating that the diseased tissue microenvironment does not affect lung-targeted carrier biodistribution. Statistical significance was determined using unpaired t-test. Data are presented as mean ± SD (*N* = 4). ns, not significant; * *p* < 0.05. Created in BioRender. Mainkar, G. (2026) https://BioRender.com/cv9279p (**A**).

To evaluate the pulmonary delivery efficacy of 50% DOTAP MC3 LNPs in a pathological context, we utilized a classical pulmonary fibrosis mouse model induced via a single IT instillation of BLM (Figure S4A). Longitudinal tracking confirmed disease development, characterized by progressive, significant body weight loss (Figure S4B) and a marked upregulation of fibrotic transcripts within lung tissue lysates (Figure S4C). To determine if intervention with LNPs interfered with disease development, we administered Luc modRNA encapsulated in 50% DOTAP MC3 LNPs via IV injection on day 10 post-injury (Figure 3A) and evaluated this cohort against mice treated with just saline or BLM. Quantitative hydroxyproline assays at day 21 verified a significant increase in total collagen content (Figure 3B), and Masson’s trichrome staining confirmed extensive tissue remodeling in both BLM-injured cohorts (Figure 3C). To determine if this severe tissue remodeling within the lungs disrupted LNP biodistribution, we performed ex vivo bioluminescence imaging on mice treated with just Luc-LNPs or BLM and Luc-LNPs on day 11. Remarkably, absolute radiance quantification demonstrated no significant differences between the two groups (Figure 3D). These findings demonstrate that the diseased tissue microenvironment does not compromise pulmonary delivery by 50% DOTAP MC3 LNPs.

### 3.4. Refinement of Pulmonary Selectivity via Endogenous MicroRNA-Mediated De-Targeting

**Figure 4:**
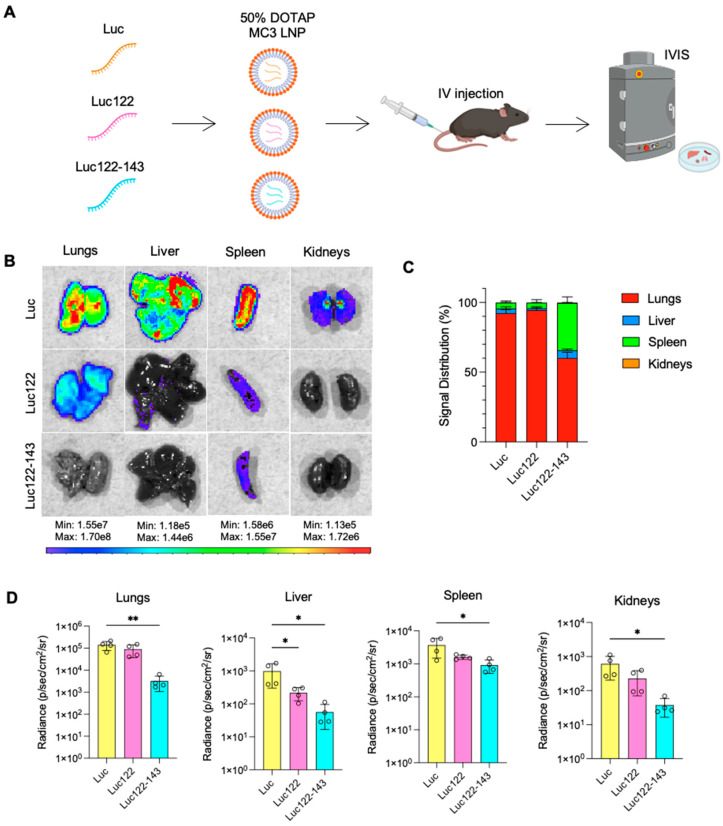
Evaluation of endogenous microRNA-mediated de-targeting to optimize lung-selective expression. (**A**). Schematic workflow of Luc, Luc122, and Luc122-143 modRNAs encapsulated in 50% DOTAP MC3 LNPs administered via IV injection, followed by ex vivo bioluminescence imaging using an IVIS. (**B**). Representative ex vivo IVIS images of lungs, liver, spleen, and kidneys from mice injected with each respective modRNA sequence. Images are shown using fixed, organ-specific intensity scales. (**C**). Relative Luc expression across organs per treatment cohort, expressed as percentage of total normalized bioluminescence signal measured across all collected organs. Data were normalized to corresponding saline control. (**D**). Quantification of ex vivo tissue bioluminescence (p/s/cm^2^/sr) across organ groups normalized to saline baseline. Statistical significance was determined using one-way ANOVA followed by Tukey’s multiple comparisons test. Data are presented as mean ± SD (*N* = 4). ns, not significant; * *p* < 0.05; ** *p* < 0.01. Created in BioRender. Mainkar, G. (2026) https://BioRender.com/3fm145i (**A**).

For further refinement of the lung-selective targeting of 50% DOTAP MC3 LNPs and the elimination of residual off-target expression, we integrated recognition sites for endogenous miRNAs into the RNA payload to leverage miRNA-mediated de-targeting strategies. We engineered two distinct Luc modRNA variants: Luc122-containing binding sites for the liver-specific miR-122, and Luc122-143-containing tandem binding sites for both miR-122 and miR-143, the latter being highly enriched within peripheral organs including the spleen, kidneys, and gastrointestinal tract [26]. These were evaluated against standard Luc as a control (Figure 4A). Following their encapsulation in LNPs and IV administration, ex vivo bioluminescence imaging revealed that while the standard Luc control mediated significant expression in both the lungs and the liver, the Luc122 construct significantly and selectively diminished the liver signal (Figure 4B,C). Conversely, while the dual-targeted Luc122-143 variant achieved complete suppression of off-target signals in the spleen, kidneys, and liver, it also led to a significant, unfavorable reduction in expression within the lungs (Figure 4B,D). Consequently, single-miRNA filtering via miR-122 emerged as the optimal de-targeting strategy for enhancing the lung tropism of the 50% DOTAP MC3 LNPs.

### 3.5. Screening of Candidate Therapeutic Genes in an In Vitro Model of TGF-β-Induced Fibrosis

**Figure 5:**
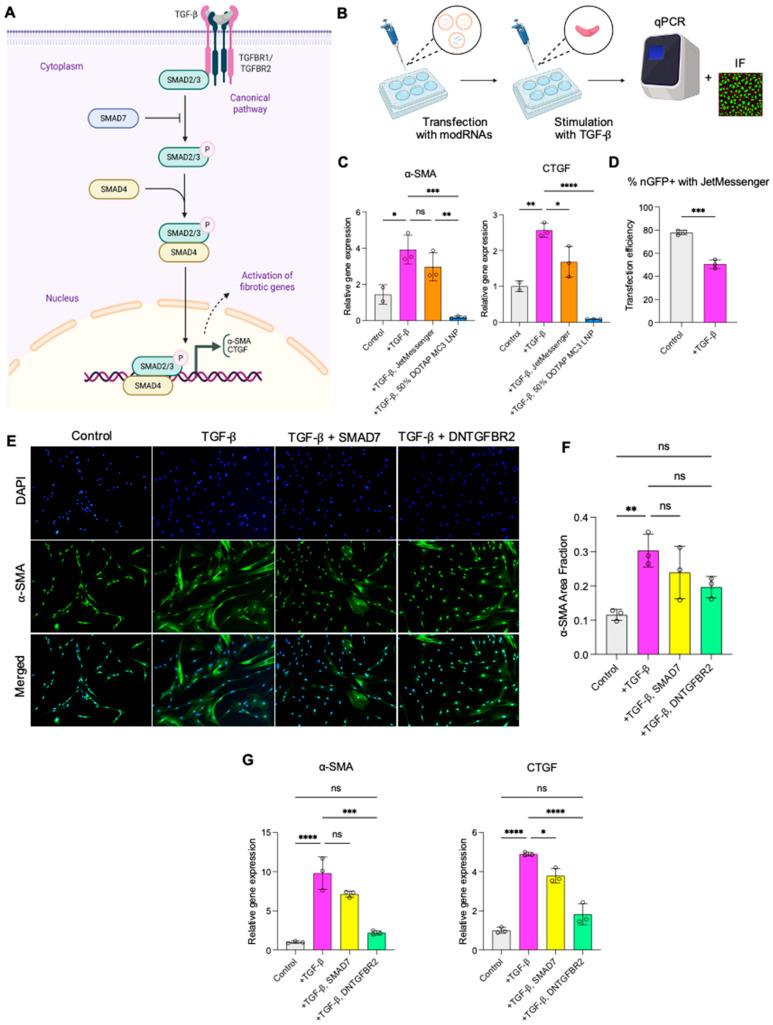
Evaluation of candidate therapeutic genes in an in vitro model of TGF-β-induced fibrosis. (**A**). Schematic overview of the canonical TGF-β pathway driving pulmonary fibrosis. Binding of TGF-β to its transmembrane receptors triggers a downstream pathway that activates primary fibrotic targets α-SMA and CTGF. Candidate therapeutic modRNAs SMAD7 and DNTGFBR2 were selected to inhibit this signaling cascade. (**B**). Schematic detailing the experimental workflow: NHLFs were transfected with modRNAs, followed by stimulation with TGF-β and subsequent gene expression analysis by qPCR and IF. Control cells were untreated and received neither TGF-β stimulation nor modRNA transfection. (**C**). Relative gene expression profiles of fibrotic markers. NHLFs demonstrated high sensitivity and fibrotic baseline disruption when treated with 50% DOTAP MC3 LNPs, necessitating the use of JetMessenger for in vitro therapeutic evaluation. (**D**). Transfection efficiency evaluated via % nGFP^+^ cells. A significant reduction in transfection efficiency was noted under TGF-β treatment conditions. (**E**). Representative IF images of NHLFs stained for nuclei (DAPI, blue) and α-SMA fibers (green) across treatment conditions. (**F**). Quantitative image analysis displaying α-SMA^+^ area fraction normalized to number of cells. (**G**). Relative gene expression profiles of fibrotic markers across treatment conditions; despite compromised transfection baselines under TGF-β treatment conditions, delivery of DNTGFBR2 modRNA yields a statistically significant reduction in fibrotic marker expression. Statistical significance was determined using one-way ANOVA followed by Tukey’s multiple comparisons test. Data are presented as mean ± SD (*N* = 3). ns, not significant; * *p* < 0.05; ** *p* < 0.01; *** *p* < 0.001; **** *p* < 0.0001. Created in BioRender. Mainkar, G. (2026) https://BioRender.com/6gx83x0 (**A**). Created in BioRender. Mainkar, G. (2026) https://BioRender.com/tdpqrl3 (**B**).

To establish a functional foundation for therapeutic intervention, we evaluated modRNAs designed to interrupt the canonical TGF-β signaling cascade, a primary driver of pathological myofibroblast differentiation and pulmonary fibrosis [27]. Binding of TGF-β to its transmembrane receptors triggers the phosphorylation and nuclear translocation of SMAD2/3 complexes paired with SMAD4, ultimately driving the transcriptional upregulation of primary downstream fibrotic targets, including α-smooth muscle actin (α-SMA) and connective tissue growth factor (CTGF) (Figure 5A). To block this signaling axis, we selected two candidate therapeutic modRNA payloads: SMAD7, an endogenous intracellular inhibitor of SMAD2/3 phosphorylation, and a dominant-negative variant of the TGF-β type II receptor (DNTGFBR2) engineered to contain only the extracellular TGF-β-binding domain so as to sequester ligands without triggering the downstream signaling pathway (Figure 5A).

For validation of these payloads, normal human lung fibroblasts (NHLFs) were transfected with the respective modRNAs prior to stimulation with exogenous TGF-β (Figure 5B). Initial baseline validation assays revealed that NHLFs exhibited high sensitivity and fibrotic baseline disruption when directly transfected with reporter gene nuclear green fluorescent protein (nGFP) using the 50% DOTAP MC3 LNPs in vitro, necessitating the use of the commercial reagent JetMessenger to establish a cleaner baseline for in vitro validation of therapeutic genes (Figure 5C). Notably, checking the transfection efficiency of the reporter payload under these conditions revealed a significant reduction in the percentage of nGFP^+^ cells under TGF-β stimulation, indicating that the activated myofibroblast phenotype resisted baseline transfection (Figure 5D). Despite the compromised transfection baseline, analysis of phenotypic changes via immunofluorescence (IF) staining and quantification of α-SMA area fractions showed that treatment with both SMAD7 and DNTGFBR2 modRNAs prevented the formation of dense α-SMA stress fibers, although DNTGFBR2 clearly outperformed SMAD7 (Figure 5E,F). Subsequent qPCR analysis mirrored these phenotypic changes, showing that DNTGFBR2 delivery yielded a significant and robust reduction in both α-SMA and CTGF transcript expression following TGF-β induction, outperforming the SMAD7 treatment group which showed more modest trends in suppression (Figure 5G). Taken together, these in vitro screening data identify DNTGFBR2 modRNA as the superior therapeutic payload capable of effectively blunting TGF-β-mediated fibrotic activation.

## 4. Discussion

The development of effective RNA therapies for pulmonary fibrosis has been limited by the lack of delivery systems capable of achieving robust and selective modRNA delivery and expression in the lungs following systemic administration. In this study, we established an integrated platform for the development of lung-targeted therapeutic modRNAs through comparative evaluation of the LNP composition, administration route, miRNA-mediated transgene de-targeting, and therapeutic payload screening. Our findings demonstrate that a 50% DOTAP MC3 LNP formulation enables efficient pulmonary delivery following IV administration, maintains pulmonary transfection in fibrotic lungs, and can be further enhanced through endogenous miRNA-mediated de-targeting to improve lung selectivity.

A major barrier to systemic nucleic acid delivery is the inherent liver tropism of conventional LNPs [28]. Previous studies demonstrated that incorporation of permanently cationic lipids can alter protein corona formation and redirect biodistribution toward the lungs [18]. The lead formulation identified in this study belongs to the recently developed class of selective organ targeting (SORT) nanoparticles, in which incorporation of the quaternary ammonium lipid DOTAP promotes formation of a distinct plasma protein corona, thereby redirecting LNP biodistribution from the liver toward the lungs [15,18]. The lipid components evaluated in this study were selected based on their complementary physicochemical properties: the phospholipid 9A1P9 facilitates membrane fusion and endosomal escape, 5A2-SC8 is a highly branched and biodegradable ionizable lipid that promotes membrane disruption, MC3 is a clinically validated ionizable lipid that enhances intracellular delivery, while cholesterol and DMG-PEG improve nanoparticle stability, membrane integrity, and circulation behavior [16,29,30,31]. Consistent with previous reports, all charge-modified formulations evaluated here exhibited enhanced pulmonary delivery compared with the neutral Lipid 5 control. However, unlike previous studies that primarily characterized individual formulations, our work directly compared multiple lung-targeted candidates under identical conditions. Although iPLNP generated the highest absolute pulmonary expression, its reduced EE may present challenges for clinical translation. In contrast, the 50% DOTAP MC3 formulation combined favorable physicochemical properties with strong pulmonary transfection and minimal off-target expression, supporting its translational potential. Another important finding was the broad cellular transfection profile achieved within the lungs. Because pulmonary fibrosis is driven by interactions among epithelial, endothelial, immune, and mesenchymal cell populations, broad transfection may be advantageous over highly restricted cell-specific delivery strategies [32,33]. In parallel, considerable advances have been made in alternative pulmonary RNA delivery platforms, including polymeric nanoparticles, extracellular vesicles, hybrid lipid–polymer systems, and inhalable formulations, many of which may offer advantages such as improved biocompatibility or distinct delivery characteristics [34,35,36,37]. Future studies should compare these emerging delivery technologies with the lung-selective LNPs described here to identify the optimal strategy for pulmonary RNA delivery. Regardless of the delivery vehicle employed, transcript engineering approaches such as miRNA-mediated de-targeting could be incorporated to further enhance tissue selectivity and enable precise deployment of disease-specific therapeutic payloads.

Comparison of administration routes further highlighted the need to balance between delivery efficiency and safety. IT administration produced highly localized pulmonary expression, consistent with previous reports of airway-directed delivery, but resulted in substantial mortality and tissue damage in our study [38]. In contrast, IV administration achieved robust pulmonary transfection while maintaining a favorable safety profile. These findings support IV administration as the preferred route for delivery of the charged lung-targeted LNPs evaluated in this study. Nevertheless, the mechanisms underlying the observed toxicity following IT administration were not investigated and warrant further study, particularly to better understand the safety profile of permanently cationic lipid-containing LNP formulations following direct pulmonary administration. In addition, recent advances in inhalable and nebulizable RNA delivery systems have demonstrated considerable promise for pulmonary therapeutics and should be directly compared with systemically administered LNPs in future studies.

The fibrotic ECM has been proposed as a barrier to nanoparticle penetration and cellular uptake [21]. Surprisingly, despite extensive tissue remodeling in the BLM mouse model, pulmonary expression levels remained unchanged following LNP administration. This suggests that lung-targeted LNP delivery is preserved even in diseased tissue. Although nanoparticle transport through fibrotic matrices has been investigated, the impact of pulmonary fibrosis on modRNA-LNP biodistribution in vivo remains poorly characterized. Our results therefore provide important evidence supporting the use of lung-targeted LNPs for therapeutic intervention in established disease. However, pulmonary delivery was evaluated at a single time point corresponding to the peak of fibrosis in the bleomycin model. Although this time point represents maximal ECM deposition, the bleomycin model undergoes spontaneous resolution following the acute injury phase and therefore does not fully recapitulate the progressive nature of human pulmonary fibrosis. Future studies should evaluate nanoparticle biodistribution and cellular targeting across multiple stages of disease progression to better understand how evolving fibrotic remodeling influences pulmonary RNA delivery. We incorporated endogenous miRNA recognition sites into the RNA payload to further improve the lung selectivity of our platform. MiRNA-mediated transgene regulation has been widely explored in viral and gene therapy applications as a strategy to improve tissue specificity but has been less extensively incorporated into pulmonary modRNA delivery platforms [39,40]. Incorporation of miR-122 target sites effectively suppressed residual hepatic expression while preserving pulmonary translation, identifying it as the optimal strategy for this platform. Future studies should determine whether hepatic miR-122 expression remains stable across different stages of pulmonary fibrosis and evaluate whether repeated or high-dose administration of miR-122-responsive modRNAs transiently alters endogenous miR-122 activity and regulates its native target transcripts. Such studies will help further optimize the safety, specificity, and long-term performance of this de-targeting strategy.

A key strength of this study is the integration of delivery optimization with therapeutic screening. Rather than focusing solely on biodistribution, we evaluated candidate antifibrotic payloads targeting the canonical TGF-β signaling pathway. TGF-β signaling is initiated through ligand binding to TGFBR2, which recruits and activates TGFBR1, leading to phosphorylation of the canonical SMAD2/3 signaling cascade. Activated SMAD2/3 complexes translocate to the nucleus, where they induce transcription of profibrotic genes involved in myofibroblast differentiation and ECM deposition, including α-SMA, CTGF, and collagen. Because dysregulated TGF-β signaling is widely recognized as a central driver of pulmonary fibrosis, multiple components of this pathway have been explored as potential therapeutic targets [23,27]. To demonstrate the utility of this in vitro screening platform for identifying promising therapeutic payloads, we selected two mechanistically distinct inhibitors of canonical TGF-β signaling. SMAD7 is an endogenous inhibitory SMAD that negatively regulates the pathway by preventing phosphorylation of SMAD2/3 and promoting degradation of activated TGF-β receptors, thereby attenuating downstream transcription of profibrotic genes [41]. In contrast, DNTGFBR2 functions at the cell surface as a dominant-negative receptor that lacks intracellular kinase activity but retains ligand-binding capability, thereby preventing activation of the endogenous TGF-β receptor complex. Both SMAD7 and DNTGFBR2 reduced fibrotic activation in NHLFs, but DNTGFBR2 produced greater suppression of the profibrotic markers α-SMA and CTGF. These findings suggest that upstream inhibition of TGF-β signaling through blockade of receptor activation may provide more effective suppression of profibrotic signaling than intracellular modulation through SMAD7 and identify DNTGFBR2 as a promising candidate for future in vivo studies.

Several alternative RNA-based therapeutic strategies have previously been investigated to attenuate TGF-β signaling, including siRNAs and ASOs targeting TGF-β ligands, receptors, or downstream SMAD proteins; oligonucleotides targeting fibrosis-associated microRNAs; and aptamers targeting TGF-β ligands, both in lung fibrosis and other disease settings [42,43,44,45,46]. These approaches primarily suppress endogenous gene expression or inhibit ligand activity and are therefore dependent on efficient knockdown or target engagement of individual pathway components. In contrast, the strategy presented here transiently expresses DNTGFBR2, a dominant-negative receptor that functions upstream of canonical signaling by preventing activation of the endogenous TGF-β receptor complex. This approach may provide more comprehensive inhibition of TGF-β signaling while retaining the transient expression profile characteristic of modRNA therapeutics. Given the complexity of pulmonary fibrosis, future studies should also evaluate combinatorial modRNA approaches targeting multiple profibrotic pathways simultaneously, as the platform developed here is readily adaptable to both individual and combination therapeutic payloads.

Several additional limitations of this study should be acknowledged. Although the lead LNP formulation was characterized in terms of particle size, polydispersity index, and encapsulation efficiency, additional physicochemical characterization, including zeta potential, nanoparticle morphology, serum stability, and RNA integrity following encapsulation, would further support translational development. Furthermore, while luciferase bioluminescence provided a functional measure of successful modRNA delivery and translation, future studies incorporating RT-qPCR quantification of tissue-associated modRNA and Western blot-based protein quantification, together with direct evaluation of intracellular trafficking and endosomal escape, would provide a more comprehensive understanding of delivery efficiency. As endosomal escape remains one of the principal bottlenecks limiting RNA therapeutics, continued optimization of this process will be critical for maximizing the therapeutic potential of pulmonary modRNA delivery. Long-term expression kinetics, repeat-dose administration, and in vivo therapeutic evaluation of DNTGFBR2 were also beyond the scope of the present study. Future investigations should additionally assess the impact of repeated administration on anti-PEG immunity, complement activation, accelerated blood clearance, and other innate immune responses to further support clinical translation. In conclusion, this work establishes a comprehensive platform for pulmonary modRNA therapeutic development. By combining lung-selective LNP engineering, optimized systemic delivery, miRNA-mediated de-targeting, and therapeutic payload screening, we provide a rational strategy for the development of next-generation RNA therapies for pulmonary fibrosis and other pulmonary diseases. Beyond pulmonary diseases, this integrated strategy provides a broadly applicable framework for the rational development of targeted RNA therapeutics, enabling systematic optimization of delivery vehicles, transcript engineering strategies, and therapeutic payloads for diseases across diverse organ systems.

## Data Availability

All modified mRNA (modRNA) vectors containing genes of interest noted in this paper will be made available to other investigators. My institution and I will adhere to the NIH Grants Policy on Sharing of Unique Research Resources including the “Sharing of Biomedical Research Resources: Principles and Guidelines for Recipients of NIH Grants and Contracts” issued in December 1999. Specifically, material transfers will be made with no more restrictive terms than in the Simple Letter Agreement or the UBMTA and without reach-through requirements. Should any intellectual property arise which requires a patent, we would ensure that the technology remains widely available to the research community in accordance with the NIH Principles and Guidelines.

## Supplementary Materials

The following supporting information can be downloaded at https://www.mdpi.com/article/10.3390/pharmaceutics18070868/s1: Table S1: Open reading frames. Figure S1: Chemical structures of lipid components used to formulate the LNPs compared for pulmonary modRNA delivery. Figure S2: Evaluation of 50% DOTAP MC3 LNP transfection efficiency across distinct pulmonary cell types in vivo. Figure S3: Evaluation of systemic toxicity and immunogenicity across various delivery routes. Figure S4: Validation of the in vivo BLM-induced pulmonary fibrosis model.

## Abbreviations

The following abbreviations are used in this manuscript:

ApoE	Apolipoprotein E
ASO	Antisense oligonucleotide
BLM	Bleomycin
CTGF	Connective tissue growth factor
Cre	Cre recombinase
DDAB	Dimethyldioctadecylammonium bromide
MC3 (DLin-MC3-DMA)	Dilinoleylmethyl-4-dimethylaminobutyrate
DNTGFBR2	Dominant-negative transforming growth factor-beta receptor II
DOTAP	1,2-Dioleoyl-3-trimethylammonium-propane
ECM	Extracellular matrix
EE	Encapsulation efficiency
FACS	Fluorescence-activated cell sorting
IF	Immunofluorescence
IP	Intraperitoneal
iPLNP	Ionizable phospholipid-based lipid nanoparticle
IT	Intratracheal
IV	Intravenous
Luc	Luciferase
LNP	Lipid nanoparticle
miRNA	MicroRNA
modRNA	Nucleoside-modified messenger RNA
NHLF	Normal human lung fibroblast
nGFP	Nuclear green fluorescent protein
PDI	Polydispersity index
qPCR	Quantitative polymerase chain reaction
RNA	Ribonucleic acid
siRNA	Small interfering RNA
SMAD	Mothers against decapentaplegic homolog
SORT	Selective organ targeting
TGF-β	Transforming growth factor-beta
TGFBR1	Transforming growth factor-beta receptor 1
TGFBR2	Transforming growth factor-beta receptor 2
α-SMA	Alpha smooth muscle actin
