# Supplementary material for: Optimization of Therapeutic modRNA Delivery to the Lung for Prevention of Pulmonary Fibrosis
Source: Pharmaceutics. 2026 Jul 16;18(7):868. doi: 10.3390/pharmaceutics18070868 (PMC13415190; doi:10.3390/pharmaceutics18070868)
Supplement: Supplementary file 1 [file pharmaceutics-18-00868-s001.zip › pharmaceutics-4398790-supplementary.pdf]

Supplementary Materials

Table S1: Open reading frames.

Luc	atggccgatgctaagaacattaagaagggccctgctcccttctaccctctggaggatggcaccgctggcgagcagctgc<br>acaaggccatgaagaggtatgccctggtgcctggcaccattgccttcaccgatgcccacattgaggtggacatcacctat<br>gccgagtacttcgagatgtctgtgcgcctggccgaggccatgaagaggtacggcctgaacaccaaccaccgcatcgtg<br>gtgtgctctgagaactctctgcagttcttcatgccagtgctgggcgccctgttcatcggagtggccgtggcccctgctaacg<br>acatttacaacgagcgcgagctgctgaacagcatgggcatttctcagcctaccgtggtgttcgtgtctaagaagggcctg<br>cagaagatcctgaacgtgcagaagaagctgcctatcatccagaagatcatcatcatggactctaagaccgactaccag<br>ggcttccagagcatgtacacattcgtgacatctcatctgcctcctggcttcaacgagtacgacttcgtgccagagtctttcga<br>cagggacaaaaccattgccctgatcatgaacagctctgggtctaccggcctgcctaagggcgtggccctgcctcatcgc<br>accgcctgtgtgcgcttctctcacgcccgcgaccctattttcggcaaccagatcatccccgacaccgctattctgagcgtg<br>gtgccattccaccacggcttcggcatgttcaccaccctgggctacctgatttgcggctttcgggtggtgctgatgtaccgctt<br>cgaggaggagctgttcctgcgcagcctgcaagactacaaaattcagtctgccctgctggtgccaaccctgttcagcttctt<br>cgctaagagcaccctgatcgacaagtacgacctgtctaacctgcacgagattgcctctggcggcgccccactgtctaag<br>gaggtgggcgaagccgtggccaagcgctttcatctgccaggcatccgccagggctacggcctgaccgagacaacca<br>gcgccattctgattaccccagagggcgacgacaagcctggcgccgtgggcaaggtggtgccattcttcgaggccaagg<br>tggtggacctggacaccggcaagaccctgggagtgaaccagcgcggcgagctgtgtgtgcgcggccctatgattatgt<br>ccggctacgtgaataaccctgaggccacaaacgccctgatcgacaaggacggctggctgcactctggcgacattgcct<br>actgggacgaggacgagcacttcttcatcgtggaccgcctgaagtctctgatcaagtacaagggctaccaggtggcccc<br>agccgagctggagtctatcctgctgcagcaccctaacattttcgacgccggagtggccggcctgcccgacgacgatgcc<br>ggcgagctgcctgccgccgtcgtcgtgctggaacacggcaagaccatgaccgagaaggagatcgtggactatgtggc<br>cagccaggtgacaaccgccaagaagctgcgcggcggagtggtgttcgtggacgaggtgcccaagggcctgaccgg<br>caagctggacgcccgcaagatccgcgagatcctgatcaaggctaagaaaggcggcaagatcgccgtgtaa
Cre	atgtccaatttactgaccgtacaccaaaatttgcctgcattaccggtcgatgcaacgagtgatgaggttcgcaagaacctg<br>atggacatgttcagggatcgccaggcgttttctgagcatacctggaaaatgcttctgtccgtttgccggtcgtgggcggcat<br>ggtgcaagttgaataaccggaaatggtttcccgcagaacctgaagatgttcgcgattatcttctatatcttcaggcgcgcgg<br>tctggcagtaaaaactatccagcaacatttgggccagctaaacatgcttcatcgtcggtccgggctgccacgaccaagtg<br>acagcaatgctgtttcactggttatgcggcggatccgaaaagaaaacgttgatgccggtgaacgtgcaaaacaggctct<br>agcgttcgaacgcactgatttcgaccaggttcgttcactcatggaaaatagcgatcgctgccaggatatacgtaatctggc<br>atttctggggattgcttataacaccctgttacgtatagccgaaattgccaggatcagggttaaagatatctcacgtactgac<br>ggtgggagaatgttaatccatattggcagaacgaaaacgctggttagcaccgcaggtgtagagaaggcacttagcctg<br>ggggtaactaaactggtcgagcgatggatttccgtctctggtgtagctgatgatccgaataactacctgttttgccgggtca<br>gaaaaaatggtgttgccgcgccatctgccaccagccagctatcaactcgcgccctggaagggatttttgaagcaactcat<br>cgattgatttacggcgctaaggatgactctggtcagagatacctggcctggtctggacacagtgcccgtgtcggagccgc<br>gcgagatatggcccgcgctggagtttcaataccggagatcatgcaagctggtggctggaccaatgtaaatattgtcatga<br>actatatccgtaacctggatagtgaaacaggggcaatggtgcgcctgctagaagatggcgattag
nGFP	atggtgagcaagggcgaggagctgttcaccggggtggtgcccatcctggtcgagctggacggcgacgtaaacggcca<br>caagttcagcgtgtccggcgagggcgagggcgatgccacctacggcaagctgaccctgaagttcatctgcaccaccg<br>gcaagctgcccgtgccctggcccaccctcgtgaccaccctgacctacggcgtgcagtgcttcagccgctaccccgacc<br>acatgaagcagcacgacttcttcaagtccgccatgcccgaaggctacgtccaggagcgcaccatcttcttcaaggacg<br>acggcaactacaagacccgcgccgaggtgaagttcgagggcgacaccctggtgaaccgcatcgagctgaagggca<br>tcgacttcaaggaggacggcaacatcctggggcacaagctggagtacaactacaacagccacaacgtctatatcatg<br>gccgacaagcagaagaacggcatcaaggtgaacttcaagatccgccacaacatcgaggacggcagcgtgcagctc<br>gccgaccactaccagcagaacacccccatcggcgacggccccgtgctgctgcccgacaaccactacctgagcaccc<br>agtccgccctgagcaaagaccccaacgagaagcgcgatcacatggtcctgctggagttcgtgaccgccgccgggatc<br>actctcggcatggacgagctgtacaagggagatccaaaaaagaagagaaaggtaggcgatccaaaaaagaagag<br>aaaggtaggtgatccaaaaaagaagagaaaggtataa
SMAD7	atgttcaggaccaaacgatctgcgctcgtccggcgtctctggaggagccgtgcgcccggcggcgaggacgaggagg<br>agggcgtgggggtggcggcggaggaggcgagctgcggggagaaggggcgacggacggccgggcttatggggct<br>ggtggcggcggtgcgggcagggctggctgctgcctgggcaaggcagtccgaggtgccaaaggtcaccaccatcccc<br>atcccccaacctcgggtgccggggcggccgggggcgccgaggcggatctgaaggcgctcacgcactcggtgctcaa<br>gaaactcaaggagcggcagctggagctgctgcttcaggccgtggagtcccgcggcggtacgcgcaccgcgtgcctcc<br>tgctgcccggccgcctggactgcaggctgggcccgggggcgcccgccagcgcgcagcccgcgcagccgccctcgtc

	ctactcgctccccctcctgctgtgcaaagtgttcaggtggccggatctcaggcattcctcggaagtcaagaggctgtgttgc tgtgaatcttacgggaagatcaaccccgagctggtgtgctgcaacccccatcaccttagtcgactctgtgaactagagtct ccccctcctccttactccagatacccaatggattttctcaaaccaactgcaggctgtccagatgctgtaccttcctccgcgg aaaccgggggaacgaattatctggcccctgggggctttcagattcccaacttcttctggagcctggggatcggtcacac tggtgcgtggtggcatactgggaggagaagactcgcgtggggaggctctactgtgtccaagagccctccctggatatctt ctatgatctacctcaggggaatggcttttgcctcggacagctcaattcggacaacaagagtcagctggtacagaaagtgc ggagcaagatcggctgtggcatccagctgacgcgggaagtggatggcgtgtgggtttacaaccgcagcagttacccca tcttcatcaagtccgccacactggacaacccggactccaggacgctgttggtgcacaaagtgttccctggtttctccatca aggcttttgactatgagaaagcctacagcctgcagcggcccaatgaccacgagttcatgcagcaaccatggacgggttt caccgtgcagatcagctttgtgaagggctggggccagtgctacacccgccagttcatcagcagctgcccgtgctggctg gaggtcatcttcaacagccggtag
DNTGFBR2	atgggtcgggggctgctcaggggcctgtggccgctgcacatcgtcctgtggacgcgtatcgccagcacgatcccaccg cacgttcagaagtcggttaataacgacatgatagtcactgacaacaacggtgcagtcaagtttccacaactgtgtaaattt tgtgatgtgagattttccacctgtgacaaccagaaatcctgcatgagcaactgcagcatcacctccatctgtgagaagcc acaggaagtctgtgtggctgtatggagaaagaatgacgagaacataacactagagacagtttgccatgaccccaagct cccctaccatgactttattctggaagatgctgcttctccaaagtgcattatgaaggaaaaaaaaaagcctggtgagactttc ttcatgtgttcctgtagctctgatgagtgcaatgacaacatcatcttctcagaagaatataacaccagcaatcctgacttgttg ctagtcatatttcaagtgacaggcatcagcctcctgccaccactgggagttgccatatctgtcatcatcatcttctactgctac cgcgttggatccatggactacaaagacgatgacgataaatag

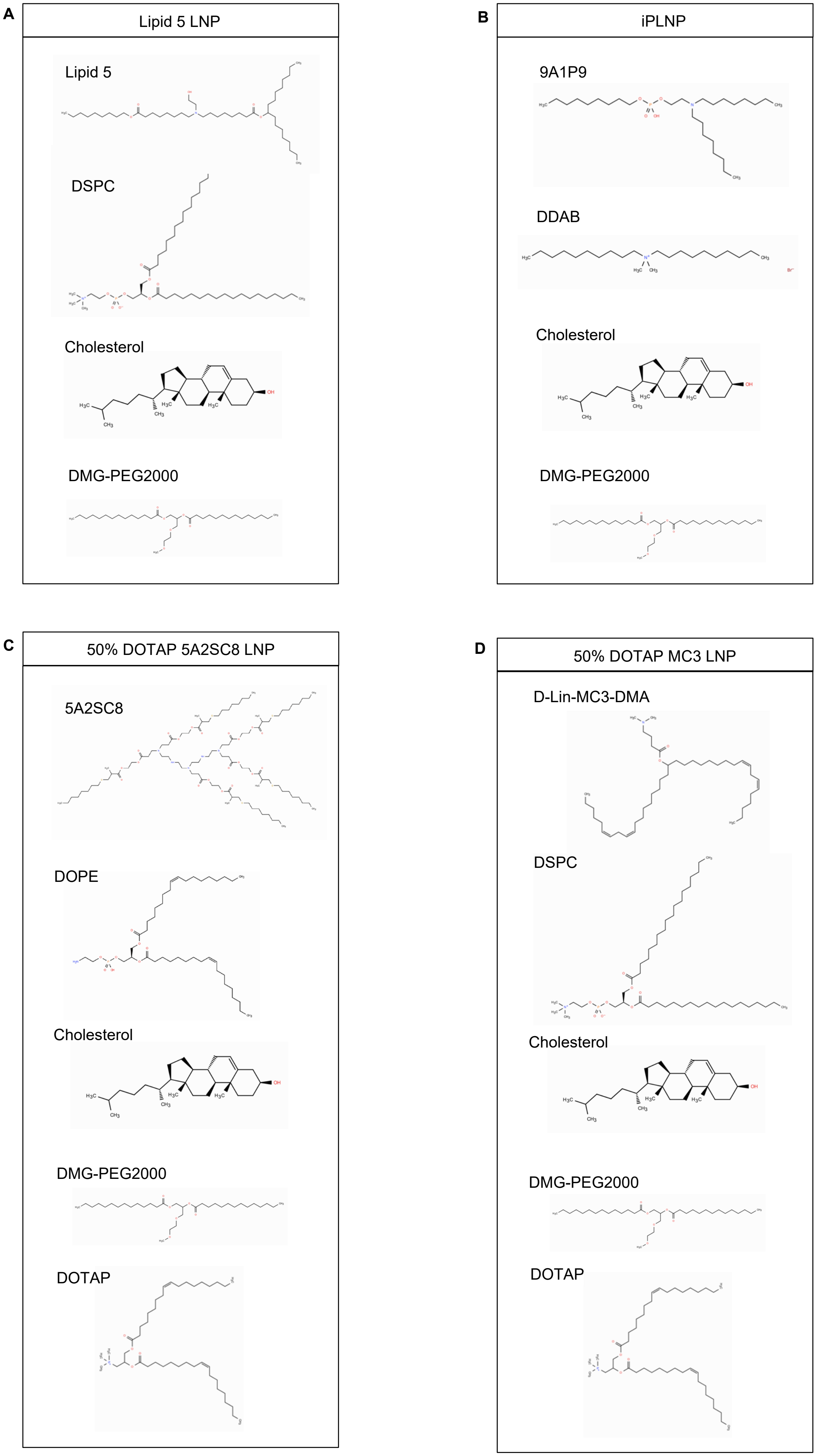
A
Lipid 5 LNP
Lipid 5
DSPC
Cholesterol
DMG-PEG2000
B
iPLNP
9A1P9
DDAB
Cholesterol
DMG-PEG2000
C
50% DOTAP 5A2SC8 LNP
5A2SC8
DOPE
Cholesterol
DMG-PEG2000
DOTAP
D
50% DOTAP MC3 LNP
D-Lin-MC3-DMA
DSPC
Cholesterol
DMG-PEG2000
DOTAP

Figure S1: Chemical structures of lipid components used to formulate the LNPs compared for pulmonary modRNA delivery. Shown are the chemical structures of the lipids used to prepare (**A**)**.** Lipid 5 LNPs, (**B**)**.** iPLNPs, (**C**)**.** 50% DOTAP 5A2SC8 LNPs and **D.** 50% DOTAP MC3 LNPs. These formulations were compared for their physicochemical properties, biodistribution, and pulmonary transfection efficiency following systemic administration.

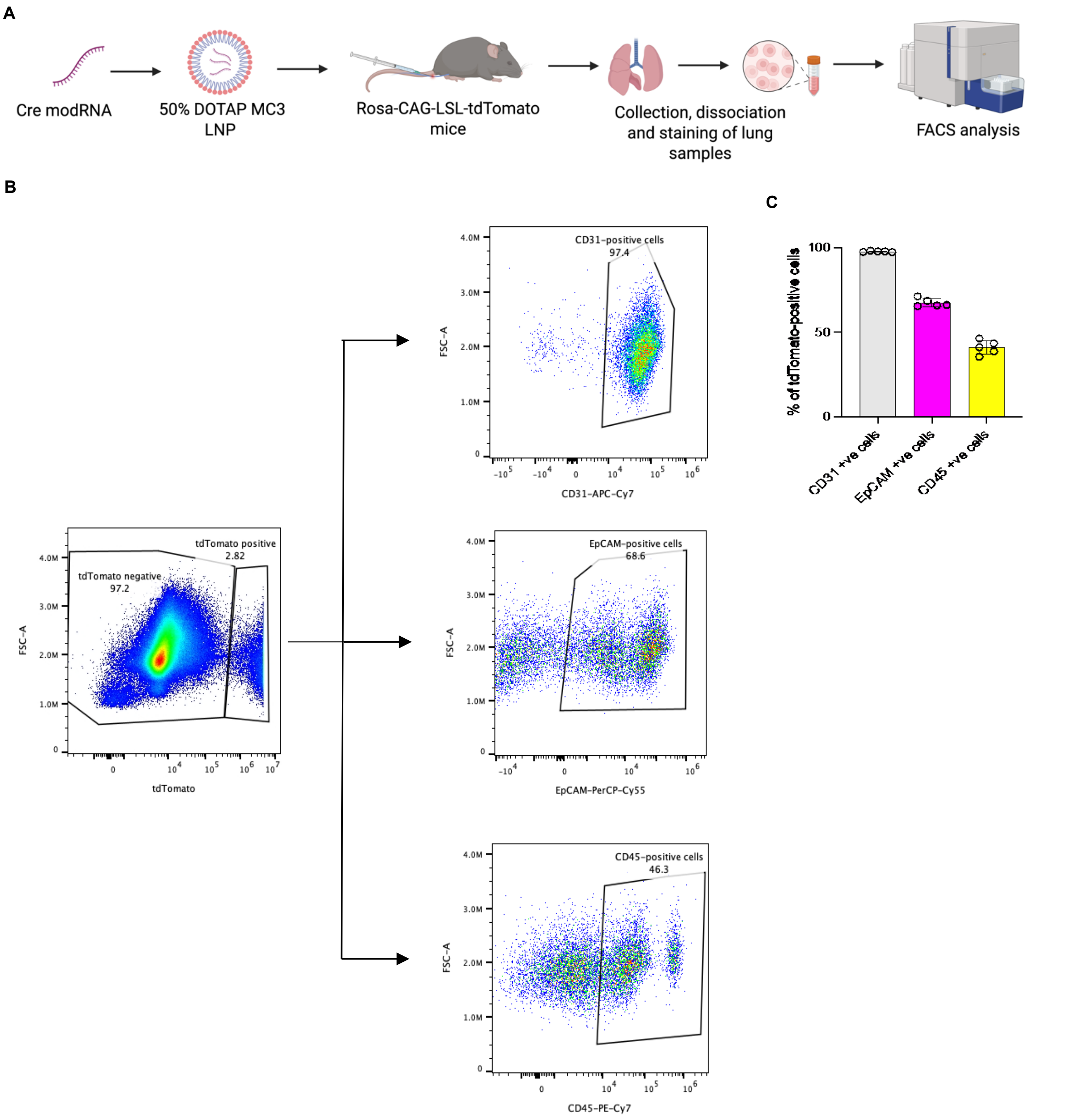

Figure S2: Evaluation of 50% DOTAP MC3 LNP transfection efficiency across distinct pulmonary cell types in vivo. (**A**). Schematic of the experimental design. Cre-dependent fluorescent reporter (*Rosa*) mice were IV injected with Cre modRNA encapsulated in 50% DOTAP MC3 LNPs, followed by lung harvest and dissociation for FACS analysis at one week post-injection. (**B**) **and** (**C**). Quantitative FACS analysis displaying the percentage of tdTomato^{+} cells across primary lung cell lineages, confirming broad transfection capability within the pulmonary microenvironment. Data are represented as mean ± SD (N = 5). Statistical significance was calculated using a one-way ANOVA.

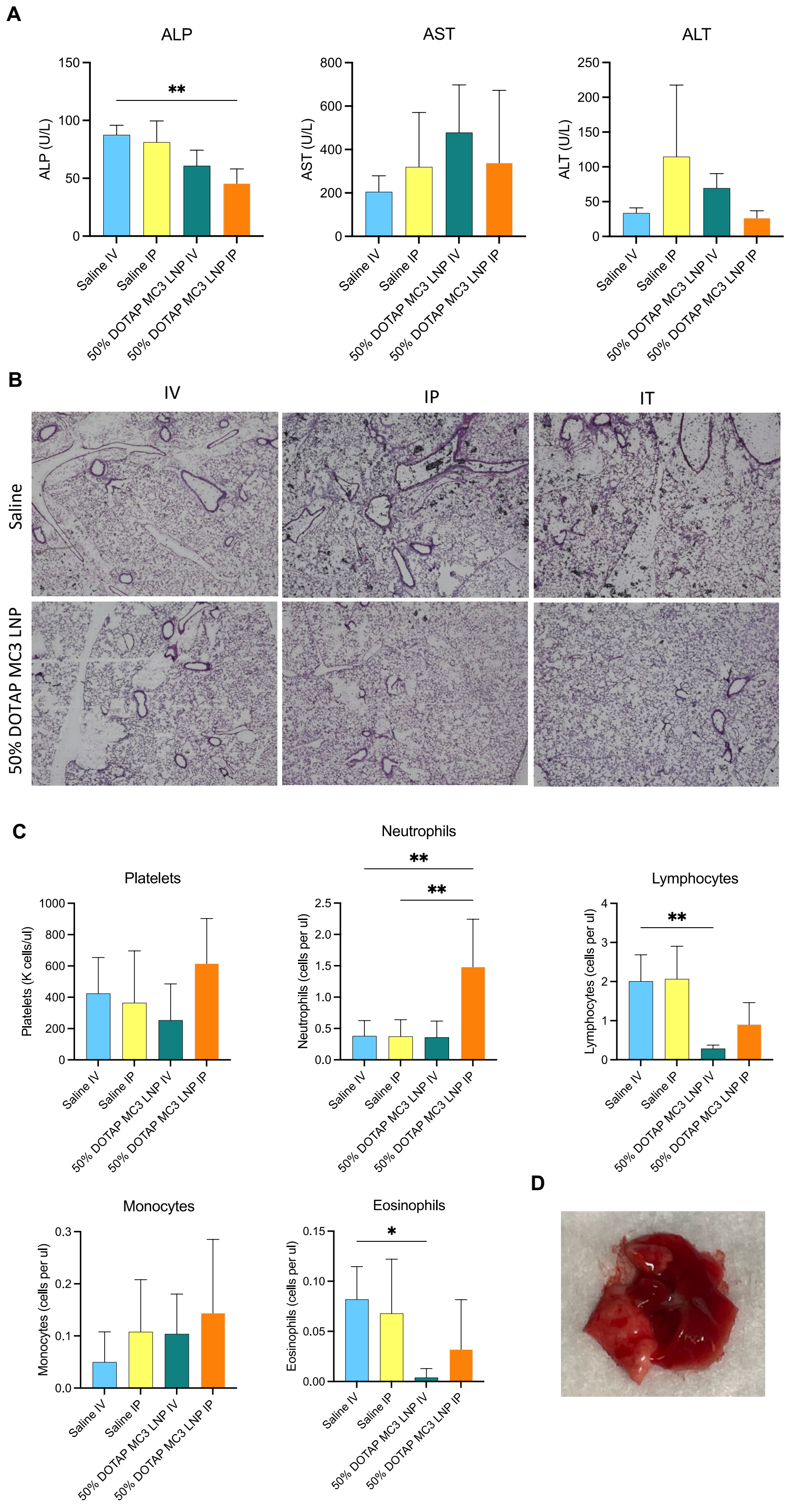

Figure S3: Evaluation of systemic toxicity and immunogenicity across various delivery routes. (**A**). Serum liver function panel comparing cohorts administered saline vs 50% DOTAP MC3 LNPs via distinct delivery routes. (**B**). Representative H&E-stained histological sections of livers from mice across cohorts showing normal liver architecture. (**C**). Profiling of immune cell counts across cohorts. IV delivery of LNPs caused a significant reduction in circulating lymphocyte and eosinophil numbers, but did not impact platelet, neutrophil, and monocyte counts. (**D**). Representative image of mouse lungs post IT instillation of LNPs. The observed severe tissue damage correlates with the high mortality of mice from this group.

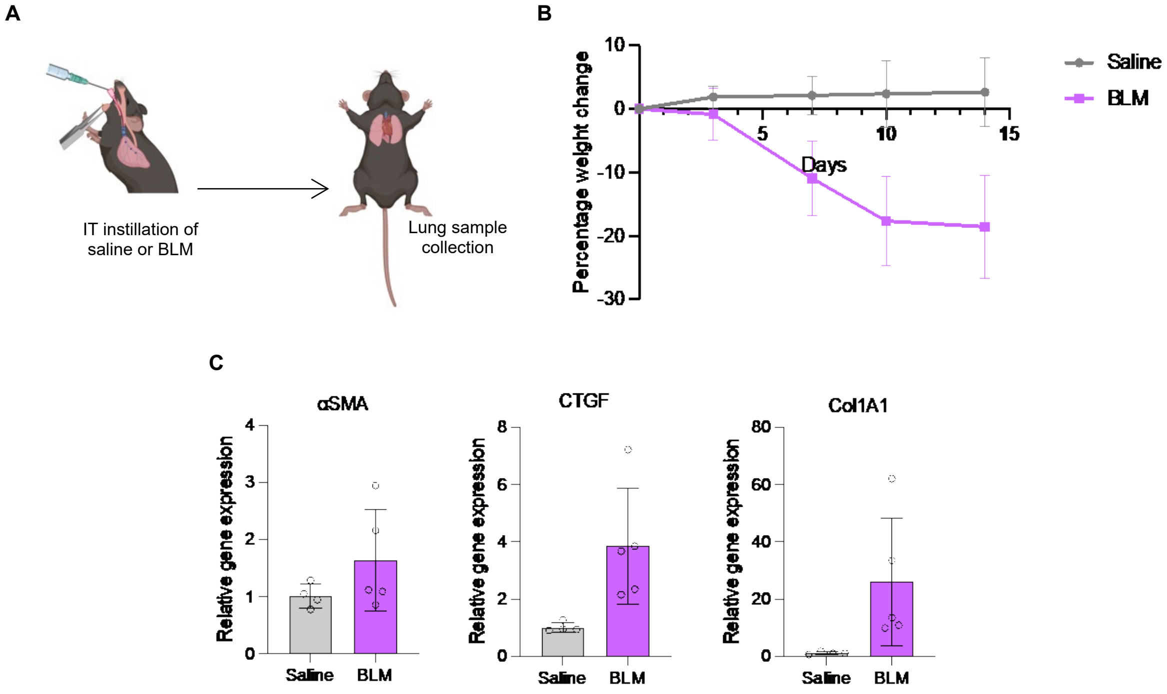

Figure S4: Validation of the in vivo BLM-induced pulmonary fibrosis model. (**A**)**.** Experimental schematic illustrating the induction of lung injury via a single IT instillation of saline in control mice or BLM in treatment mice. (**B**)**.** Longitudinal tracking of percentage body weight change across 14 days, showing a progressive weight loss characteristic of disease development. (**C**)**.** Expression analysis of hallmark fibrotic markers within lung tissue lysates via qPCR. Data are represented as mean ± SD (*N* = 4-5). Statistical significance was calculated using an unpaired t-test.
